# Supplementary figures and images for: The potential role of cuproptosis-related genes for therapy and immunoregulation in pan-cancer
Source: PLoS One. 2025 Jul 2;20(7):e0324389. doi: 10.1371/journal.pone.0324389 (PMC12220987; doi:10.1371/journal.pone.0324389)

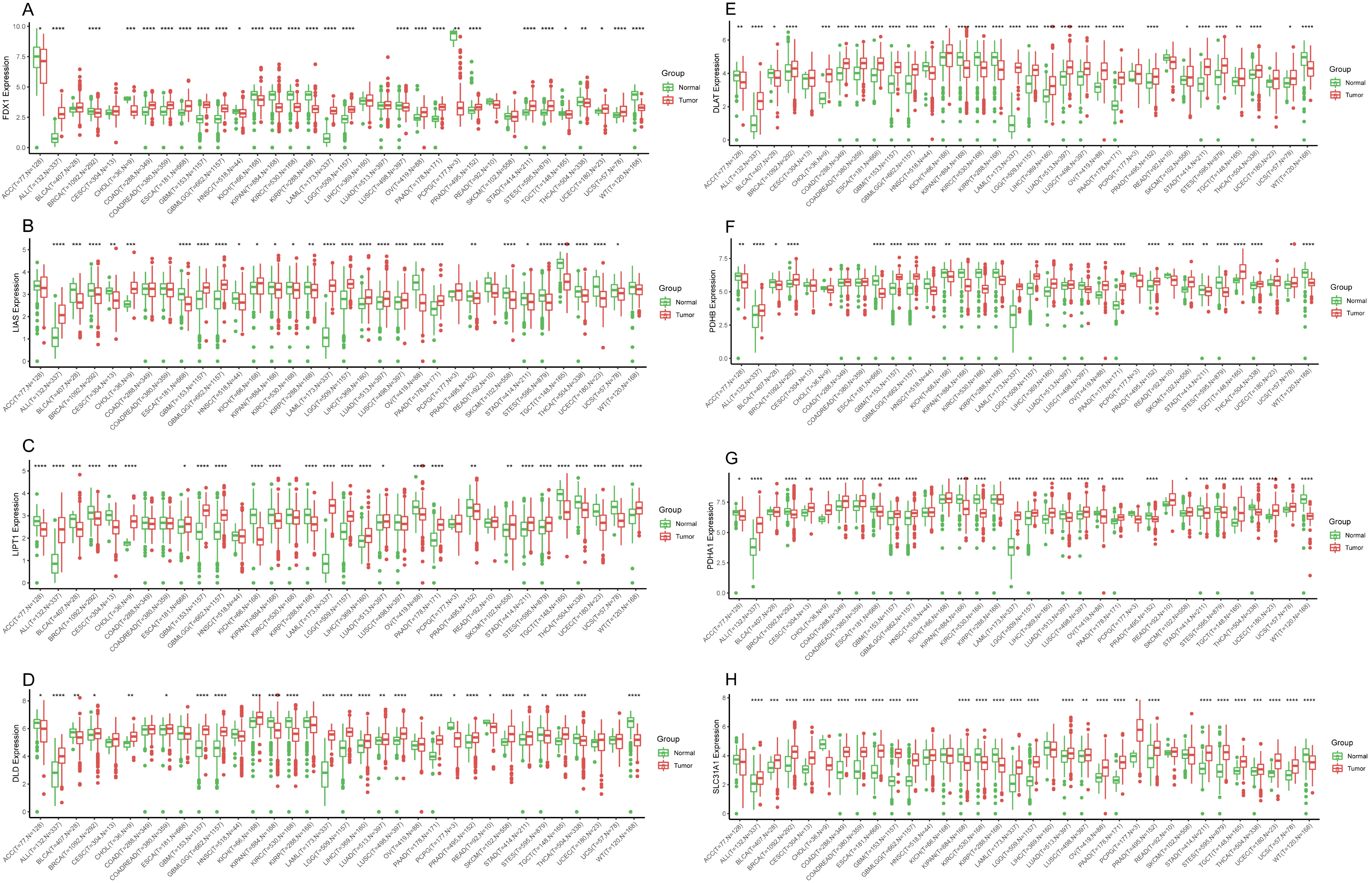

Supplement: S1 Fig — (A) FDX1 differential expression between cancer and adjacent normal tissue. (B) LIAS differential expression between cancer and adjacent normal tissue. (C) LIPT1 differential expression between cancer and adjacent normal tissue. (D) DLD differential expression between cancer and adjacent normal tissue. (E) DLAT differential expression between cancer and adjacent normal tissue. (F) PDHB differential expression between cancer and adjacent normal tissue. (G) PDHA1 differential expression between cancer and adjacent normal tissue. (H) SLC31A1 differential expression between cancer and adjacent normal tissue. (TIF) [file pone.0324389.s001.tif]

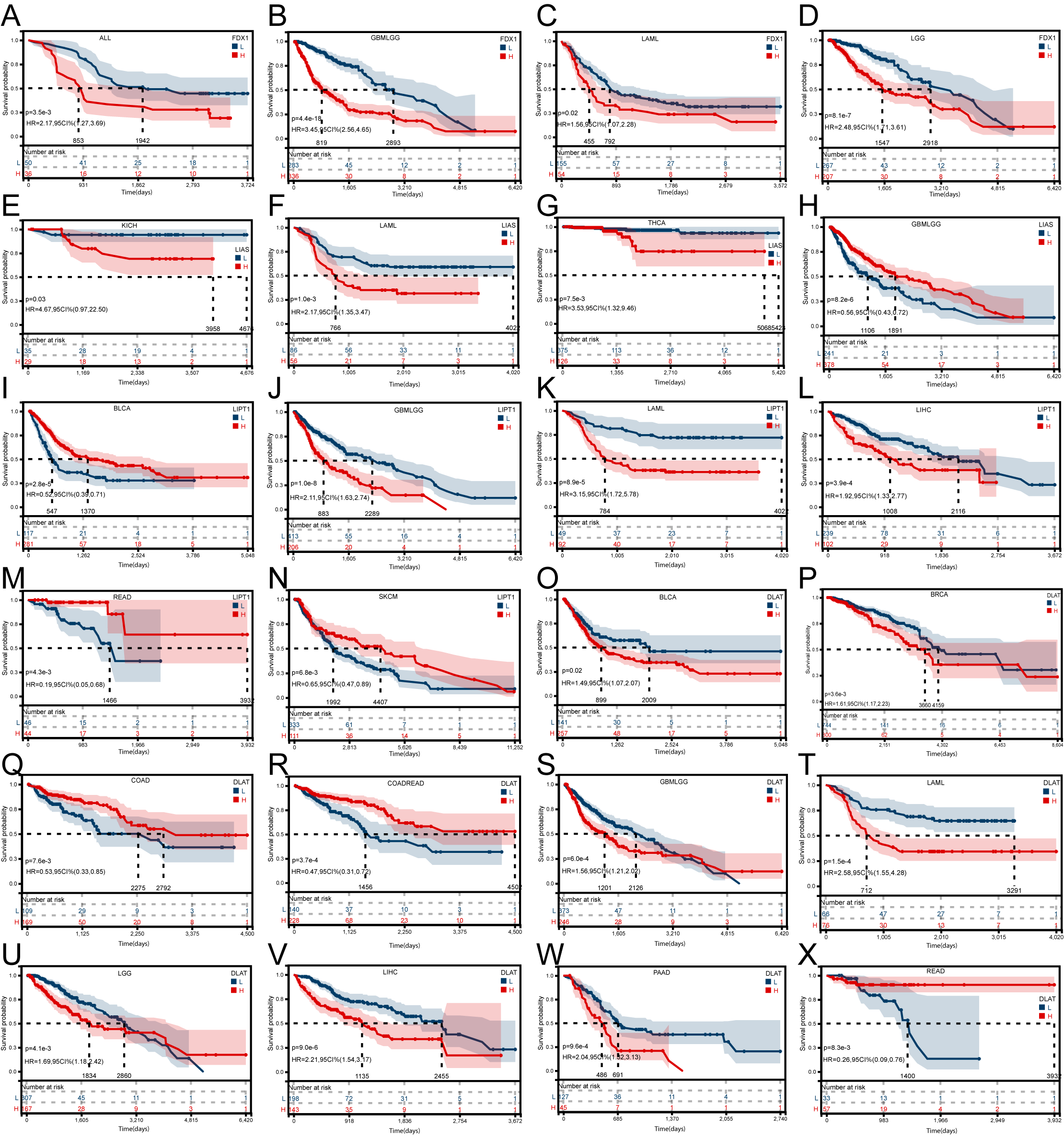

Supplement: S2 Fig — The Kaplan-Meier curves of overall survival for the expression(the median expression as a cut-off) of FDX1 in (A) ALL, (B) GBMLGG, (C) LAML, and (D) LGG. The Kaplan-Meier curves of overall survival for the expression(the median expression as a cut-off) of LIAS in (E) KICH, (F) LAML, (G) THCA, (H) GBMLGG. The Kaplan-Meier curves of overall survival for the expression(the median expression as a cut-off) of LIPT1 in (I) BLCA, (J) GBMLGG, (K) LAML, (L) LIHC, (M)READ, (N) SKCM. The Kaplan-Meier curves of overall survival for the expression(the median expression as a cut-off) of DLAT in (O)BLCA, (P) BRCA, (Q) COAD, (R) COADREAD, (S) GBMLGG, (T) LAML, (U) LGG, (V) LIHC, (W) PAAD, (X) READ. (TIF) [file pone.0324389.s002.tif]

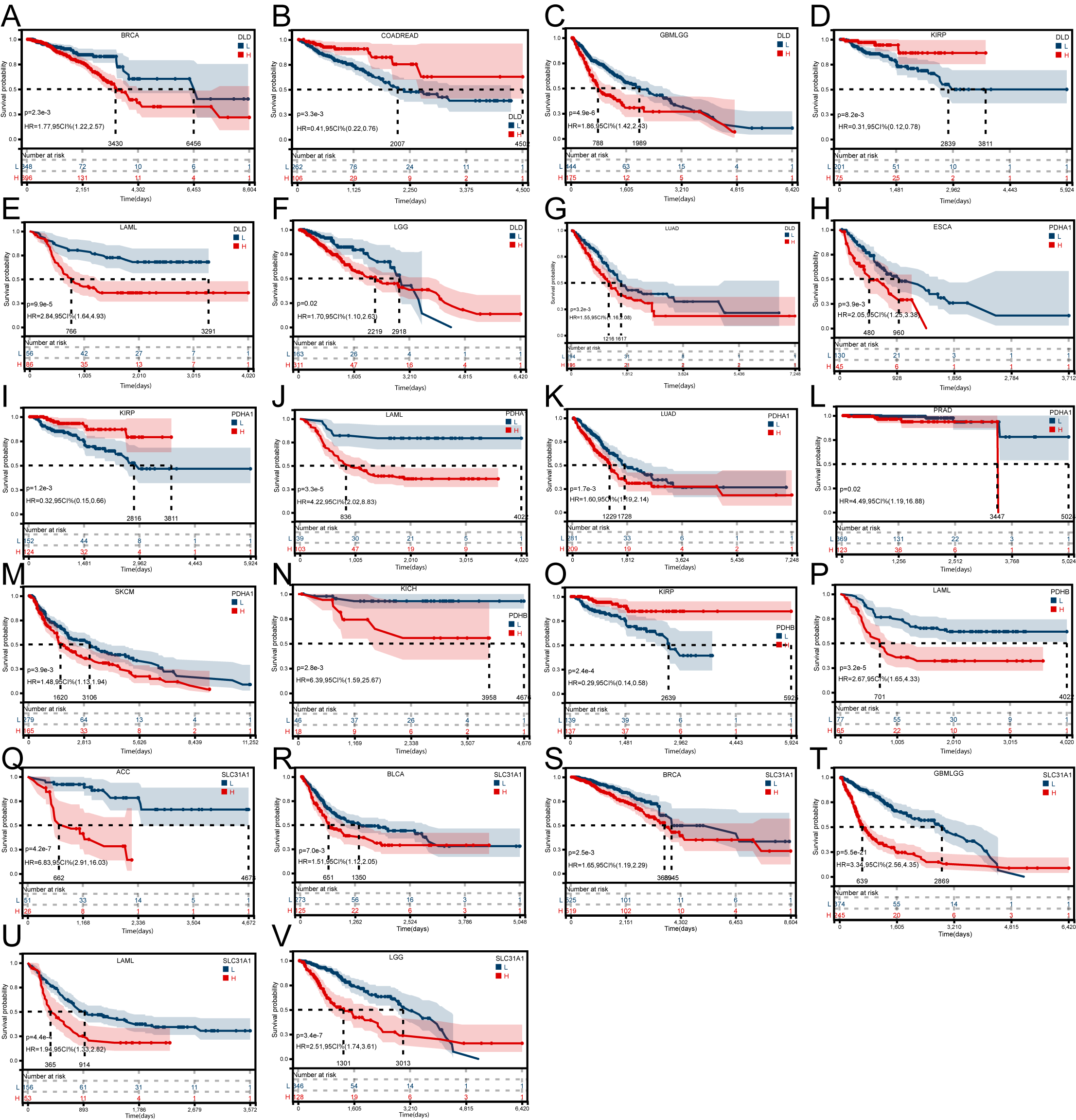

Supplement: S3 Fig — The Kaplan-Meier curves of overall survival for the expression(the median expression as a cut-off) of DLD in (A) BRCA, (B) COADREAD, (C) GBMLGG, (D) KIRP, (E)LAML, (F) LGG, (G) LUAD. The Kaplan-Meier curves of overall survival for the expression(the median expression as a cut-off) of PDHA1 in (H) ESCA, (I) KIRP, (J) LAML, (K) LUAD, (L) PRAD, (M) SKCM. The Kaplan-Meier curves of overall survival for the expression(the median expression as a cut-off) of PDHB in (N) KICH, (O) KIRP, and (P) LAML. The Kaplan-Meier curves of overall survival for the expression(the median expression as a cut-off) of SLC31A1 in (Q) ACC, (R) BLCA, (S) BRCA, (T) GBMLGG, (U) LAML, (V) LGG. (TIF) [file pone.0324389.s003.tif]

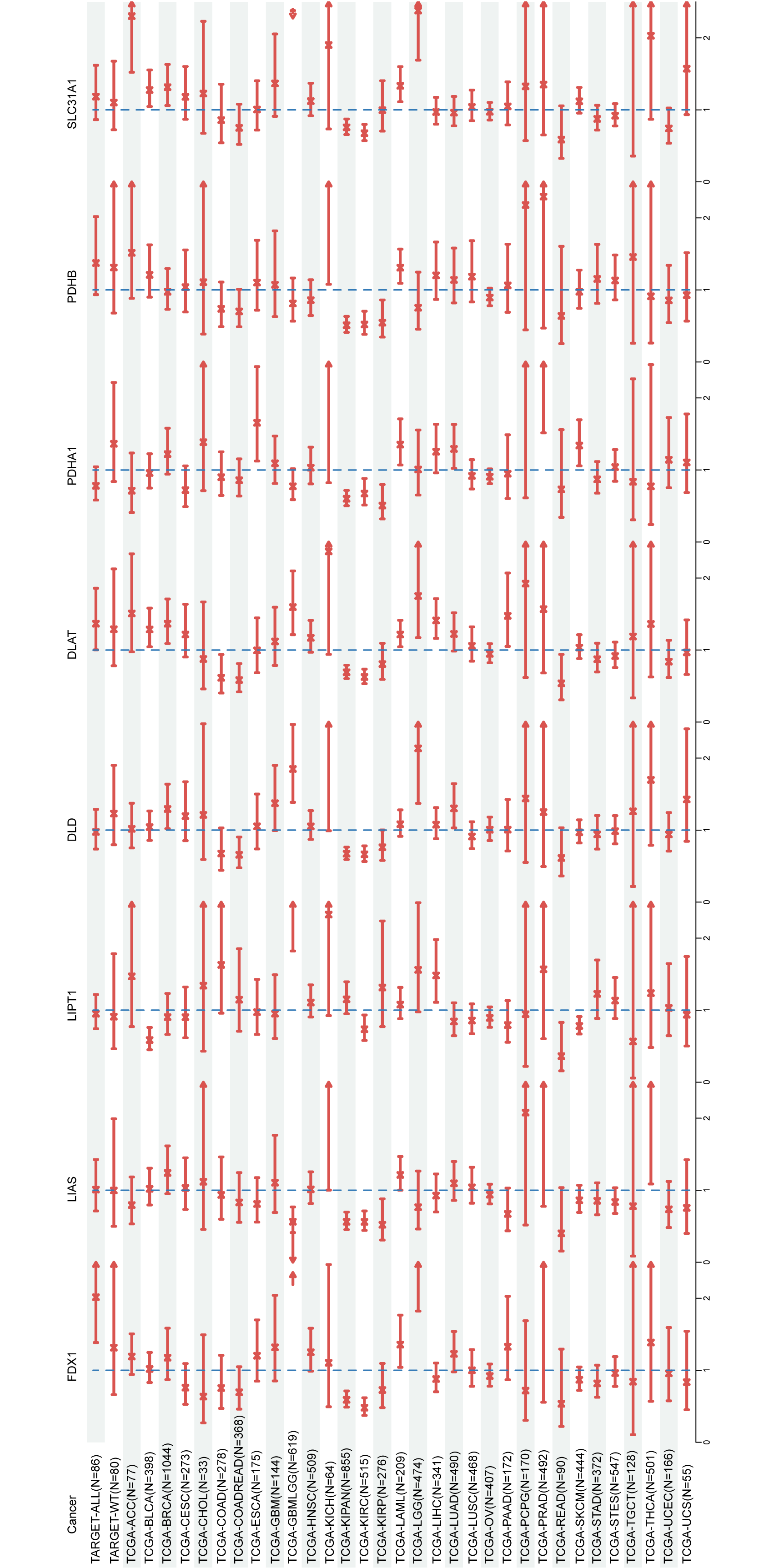

Supplement: S4 Fig — (TIF) [file pone.0324389.s004.tif]

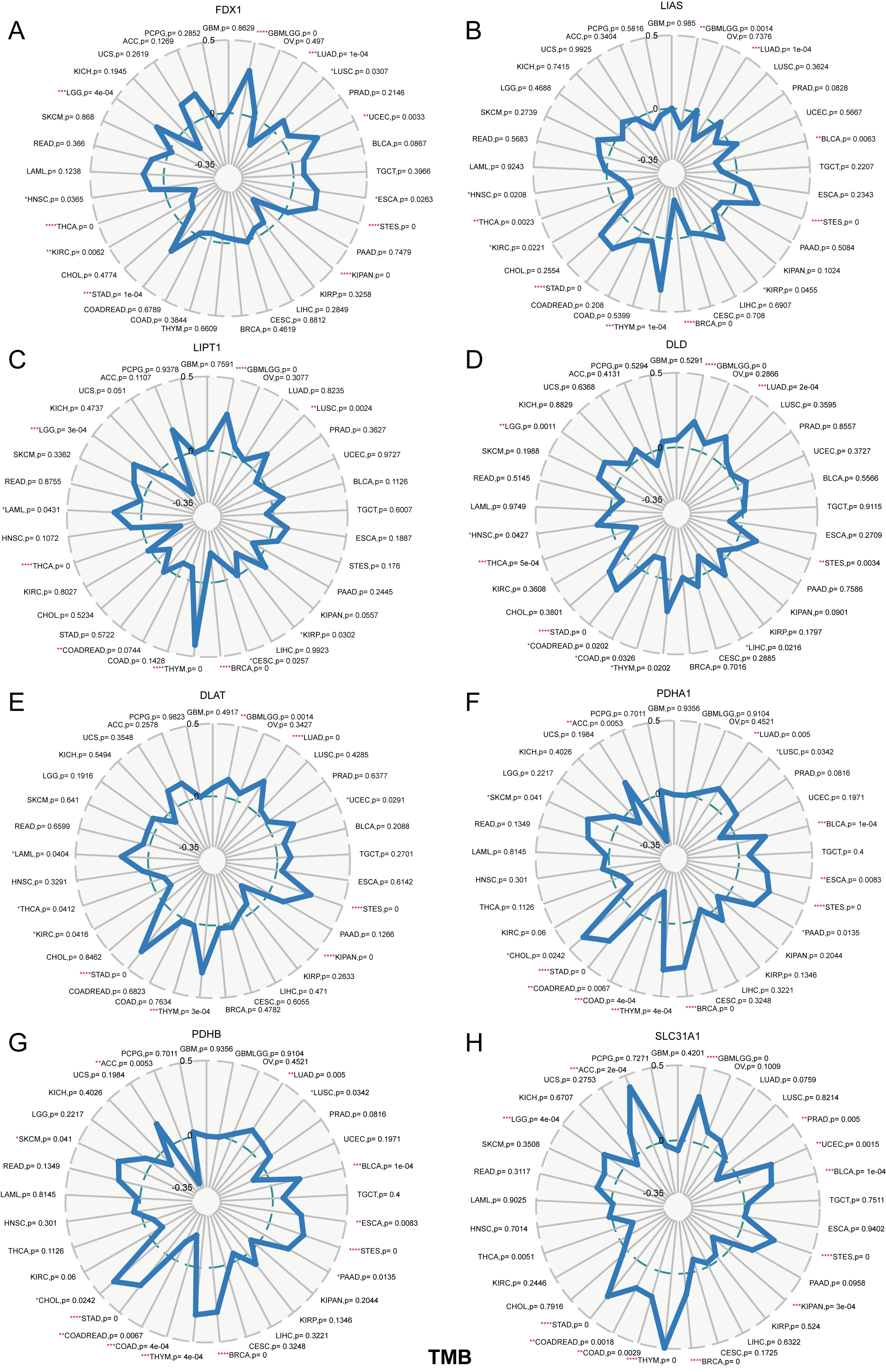

Supplement: S5 Fig — The correlations between TMB and the expression of (A) FDX1, (B) LIAS, (C) LIPT1, (D) DLD, (E) DLAT, (F) PDHA1, (G) PDHB, (H) SLC31A1. * p < 0.05, ** p < 0.01, *** p < 0.001, **** p < 0.0001. (TIF) [file pone.0324389.s005.tif]

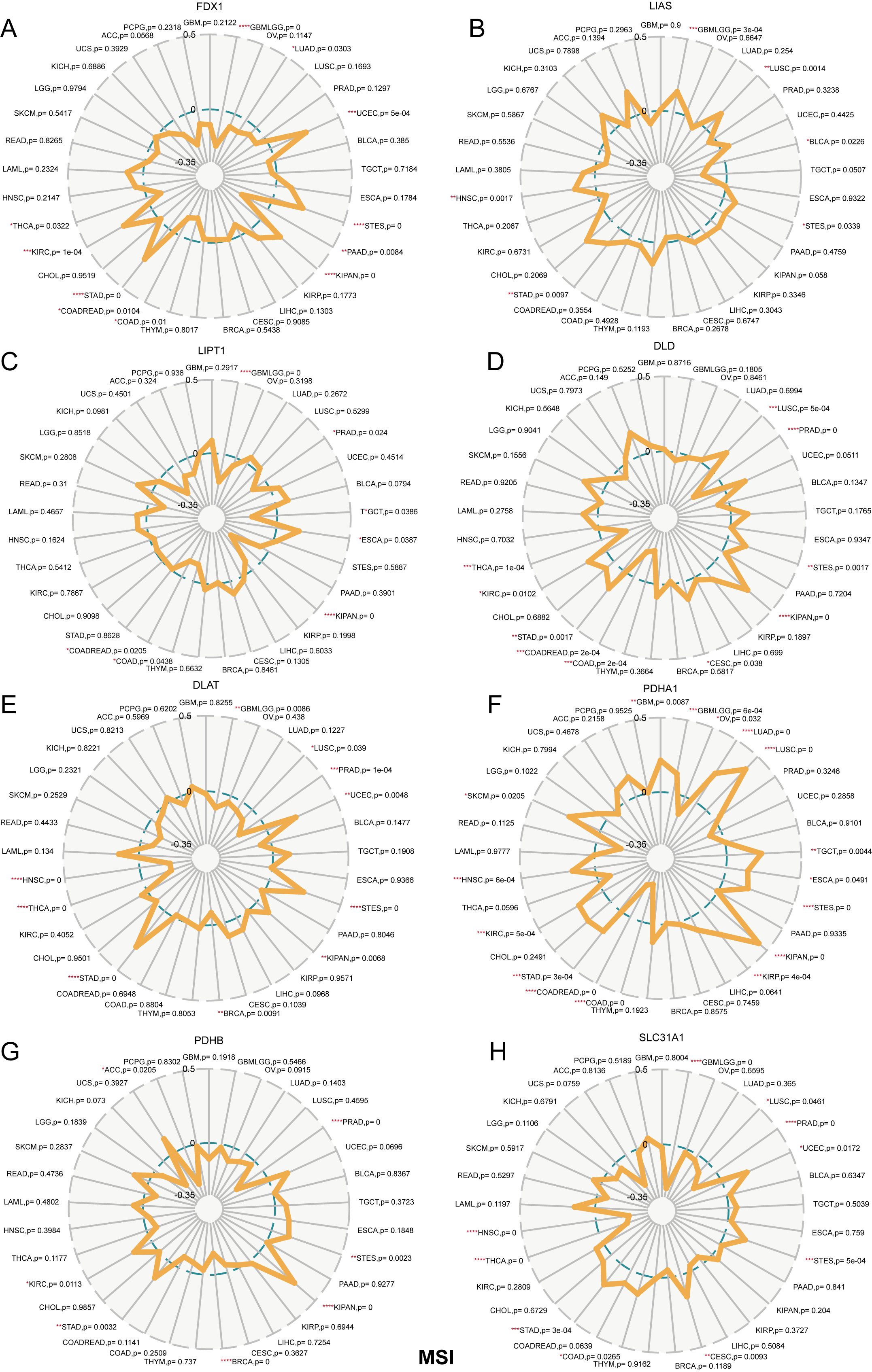

Supplement: S6 Fig — The correlations between MSI and the expression of (A) FDX1, (B) LIAS, (C) LIPT1, (D) DLD, (E) DLAT, (F) PDHA1, (G) PDHB, (H) SLC31A1. * p < 0.05, ** p < 0.01, *** p < 0.001, **** p < 0.0001. (TIF) [file pone.0324389.s006.tif]

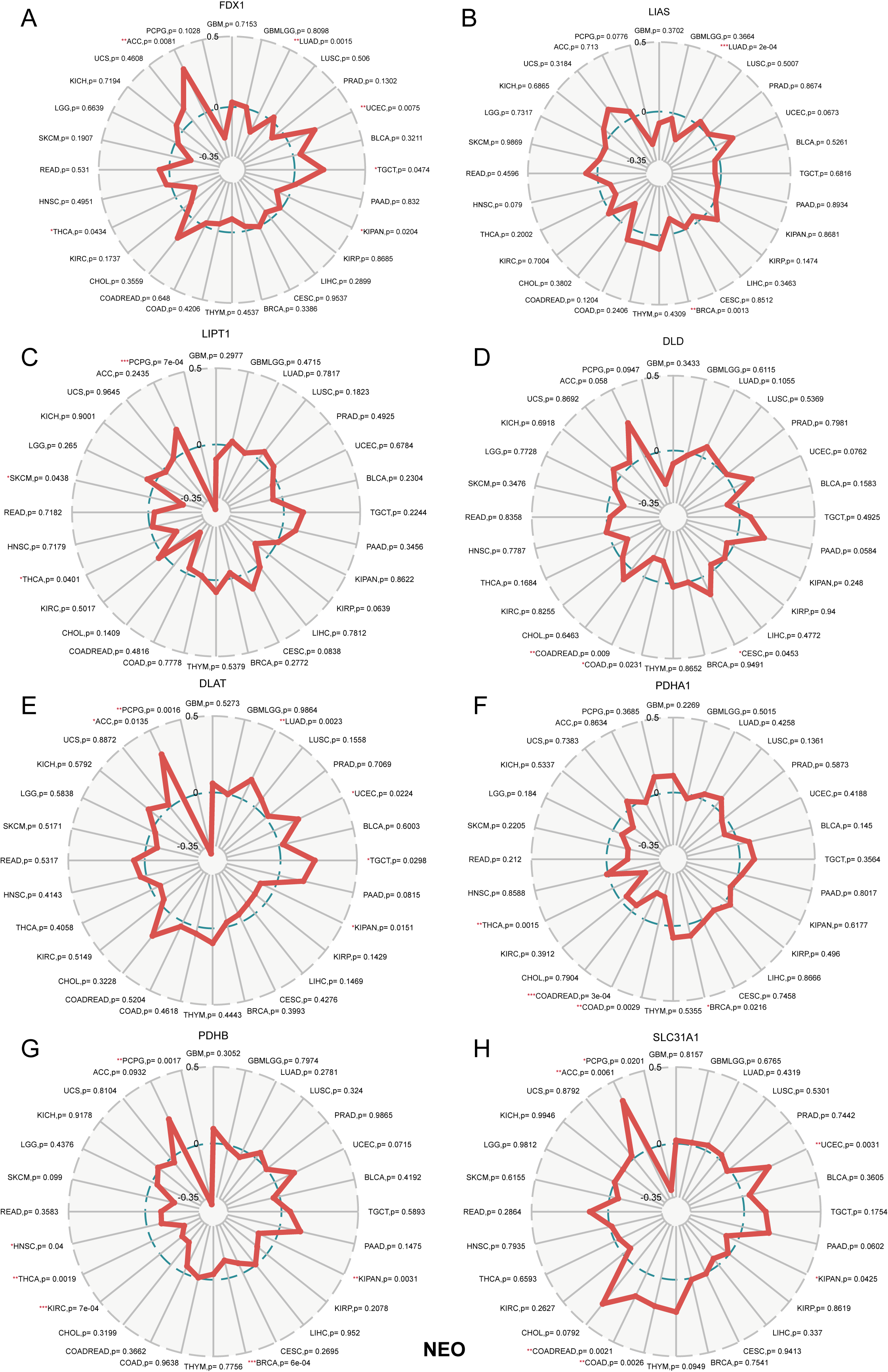

Supplement: S7 Fig — The correlations between NEO and the expression of (A) FDX1, (B) LIAS, (C) LIPT1, (D) DLD, (E) DLAT, (F) PDHA1, (G) PDHB, (H) SLC31A1. * p < 0.05, ** p < 0.01, *** p < 0.001, **** p < 0.0001. (TIF) [file pone.0324389.s007.tif]

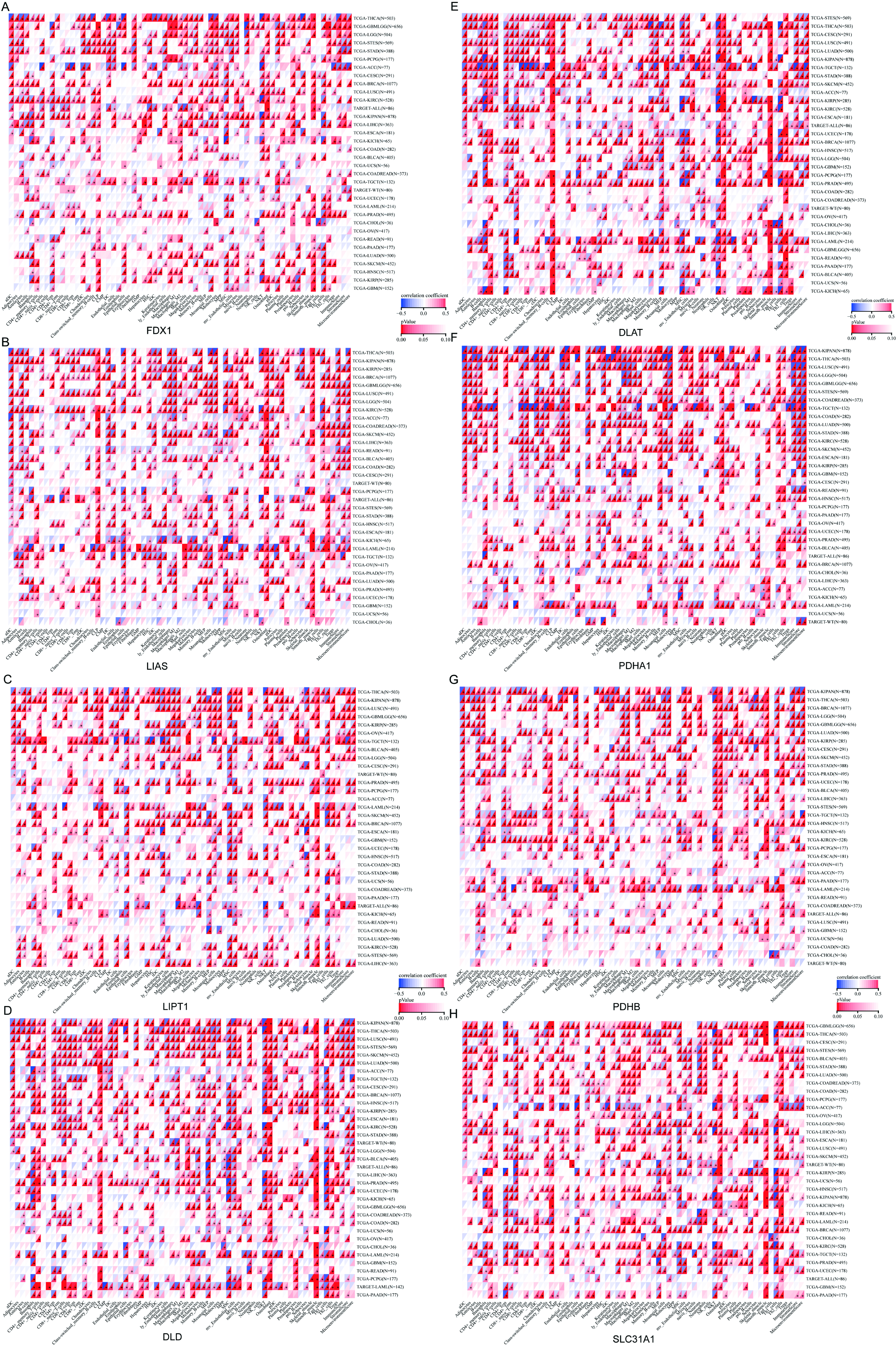

Supplement: S8 Fig — Correlations between cells infiltrating in TME of various cancer types and (A) FDX1, (B) LIAS, (C) LIPT1, (D) DLD, (E)DLAT, (F) PDHA1, (G) PDHB, (H) SLC31A1. (TIF) [file pone.0324389.s008.tif]
