# Supplementary material for: The potential role of cuproptosis-related genes for therapy and immunoregulation in pan-cancer
Source: PLoS One. 2025 Jul 2;20(7):e0324389. doi: 10.1371/journal.pone.0324389 (PMC12220987; doi:10.1371/journal.pone.0324389)
Supplement: S2 Table — (DOCX) [file pone.0324389.s010.docx]

**Supplementary Table 2. Software details**

| Software | Version | Link |
| --- | --- | --- |
| corrplot | 0.92 | https://CRAN.R-project.org/package=corrplot |
| estimate | 2.0.0 | https://bioinformatics.mdanderson.org/public-software/estimate/ |
| fmsb | 0.7.3 | https://CRAN.R-project.org/package=fmsb |
| GenVisR | 1.26.0 | https://bioconductor.org/packages/GenVisR/ |
| ggplot2 | 3.3.5 | https://CRAN.R-project.org/package=ggplot2 |
| ggpubr | 0.4.0 | https://rpkgs.datanovia.com/ggpubr |
| ggradar | 0.2 | https://github - ricardo-bion/ggradar: radar charts with ggplot2 |
| maftools | 2.12.0 | https://www.bioconductor.org/packages/release/bioc/html/maftools.html |
| pheatmap | 1.0.12 | https://CRAN.R-project.org/package=pheatmap |
| survminer | 0.4.9 | https://CRAN.R-project.org/package=survminer |
| survival | 3.3-1 | https://cran.r-project.org/package=survival |
