# Supplementary material for: The potential role of cuproptosis-related genes for therapy and immunoregulation in pan-cancer
Source: PLoS One. 2025 Jul 2;20(7):e0324389. doi: 10.1371/journal.pone.0324389 (PMC12220987; doi:10.1371/journal.pone.0324389)
Supplement: Attachment — Submitted filename: PONE-D-24-48029_R1_Response Letter.docx [file pone.0324389.s012.docx]

Dear Prof. Coller,

Thank you very much for your decision letter and advice on our manuscript (MS#PONE-D-24-48029) entitled “The potential role of cuproptosis-related genes for therapy and immunoregulation in pan-cancer”. We also thank the reviewers for the constructive comments and suggestions. We have revised the manuscript accordingly, and all amendments are indicated by red font in the revised manuscript. In addition, our point-by-point responses to the comments are listed below this letter.

We hope that our revised manuscript is now acceptable for publication in your journal and look forward to hearing from you soon.

With best wishes,

Yours sincerely,

Corresponding author

**Reviewer #1**

**Comment 1:** *All abbreviations in the Abstract should be deciphered.*
**Response:** Thank you for your suggestion. We have now spelled out all abbreviations (e.g., TMB, MSI, NEO, TME) upon their first appearance in the abstract.

**Comment 2:** *Introduction should mention existing controversies about cuproptosis role in cancers and tumour microenvironment; refer to Zhao et al., 2024.*
**Response:** We have revised the Introduction to include the controversies regarding the role of cuproptosis in the tumor microenvironment, as highlighted in the review by Zhao et al., 2024 (PMID: 39068453 ). This has been added to better contextualize our study’s focus on immunoregulation.

**Comment 3:** *Figure 2 quality is low in the PDF. Improve the resolution and font size.*
**Response:** We have regenerated Figure 2 with improved resolution and larger font size. The figure has also been processed through the PACE system to meet PLOS standards.

**Comment 4:** *There are some English mistakes. Please clarify what “drug sensibility” means.*
**Response:** Thank you for pointing this out. The term “drug sensibility” has been corrected to “drug sensitivity” throughout the manuscript.

**Comment 5:** *Line 343: revise “subsequent treatment with the mechanism of cuproptosis.”*
**Response:** This sentence has been rephrased as: “subsequent treatment using drugs that activate cuproptosis.”

**Comment 6:** *Discussion needs English editing. Line 349 and others require rewriting.*
**Response:** We have thoroughly edited the Discussion section for grammar and clarity, especially from line 349 onward. The section now uses more concise and academically appropriate phrasing.

**Comment 7:** *Extend the discussion by describing how FDX1 can be targeted and its immune-related roles.*
**Response:** Additional content has been added to the Discussion regarding the therapeutic potential of targeting FDX1, including its involvement in modulating immune cell infiltration within the tumor microenvironment.

**Comment 8:** *Focus more on promising genes. The current discussion is too general.*
**Response:** We now highlight key genes such as SLC31A1 and FDX1 in more depth, including their consistent prognostic roles and relevance for predictive scoring. A paragraph has been added to support their use in future modeling strategies.

**Reviewer #2**

**Comment 1:** *The study lacks novelty as similar work (Yang et al., 2024) already exists.*
**Response:** We appreciate this observation. While Yang et al., 2024 analyzed a broader set of genes, our study distinguishes itself by:

1. Integrating datasets from GTEx, TCGA, and TARGET;
2. Applying xCell and ESTIMATE analyses to dissect immune infiltration at high resolution;
3. Exploring drug sensitivity with FDA-approved compounds and highlighting six potential therapeutic agents;
4. Providing pan-cancer survival and staging analysis, which is more detailed than Yang’s prostate-focused validation.
   We have added clarifications in the Introduction and Discussion to better reflect this novelty.

**Comment 2:** *Lack of experimental validation limits the impact.*
**Response:** We acknowledge this limitation and now include a statement in the Discussion noting that further experimental validation in specific cancer types is essential and planned for future research.
